# Population Health, Place, and Space: Spatial Perspectives in Chronic Disease Research and Practice

**DOI:** 10.5888/pcd16.190237

**Published:** 2019-09-05

**Authors:** Michele Casper, Michael R. Kramer, James M. Peacock, Adam S. Vaughan

**Affiliations:** 1 Division for Heart Disease and Stroke Prevention, Centers for Disease Control and Prevention, Atlanta, Georgia; 2 Department of Epidemiology, Rollins School of Public Health, Emory University, Atlanta, Georgia; 3 Cardiovascular Health Unit, Minnesota Department of Health, St. Paul, Minnesota

Understanding the role of place and space in shaping the geographic distributions of chronic disease is critical to informing appropriate public health responses for chronic disease prevention and treatment. A geospatial perspective on chronic disease expands the focus of public health efforts beyond the individual, providing insights and guidance for action at the community, regional, and/or national levels. Accordingly, the articles in this special collection advance our understanding of population health dynamics and geospatial disparities for a wide range of chronic disease outcomes across 3 broad themes:

1. Examining connections between community-level characteristics and population health
2. Developing and applying spatial statistical methods and new geospatial tools
3. Using maps and geospatial results to guide program and policy decisions

## Examining Connections Between Community-Level Characteristics and Population Health

Geospatial studies are uniquely designed to examine the contextual characteristics of communities that may affect opportunities for chronic disease prevention and treatment. The contextual characteristics addressed in this collection, *Population Health, Place, and Space: Spatial Perspectives in Chronic Disease Research and Practice*, range from underlying context (such as neighborhood deprivation [1], racial segregation [2], social capital [3], and resiliency [4]) to the built environment (walkability [5,6], park access [7], and healthy restaurants [8]) and environmental exposures (9). The study comparing cardiovascular disease–resilient neighborhoods with cardiovascular disease–at-risk neighborhoods examines the important, but understudied, concept of neighborhood resiliency as it affects black populations (4). The study of neighborhood risk and pediatric asthma provides additional evidence of the need for interventions that move beyond primary care or clinical settings (1). Through their maps and spatial analyses, these studies reinforce that chronic diseases are not randomly distributed across communities, emphasize that drivers of disease occur at multiple geographic levels, and stress the importance of developing and implementing programs and policies that address the relevant contextual characteristics.

## Developing and Applying Spatial Statistical Methods and New Geospatial Tools

This is a time of great advances in the development and application of spatial statistics, spatial tools, spatially referenced data sets, and spatial data visualization — all of which enable public health professionals to more precisely understand and address existing inequities in chronic diseases. Many studies in this collection use state-of-the-art spatial statistics, including Bayesian spatial smoothing (10,11) and the spatial Durbin econometric model (3), along with other advanced spatial analytic techniques, such as hot spot analysis (12) and spatial scan statistics for spatial clustering (13), and trajectory analysis (14). Furthermore, the development of 2 spatial analysis tools is included in this collection – The Peel Walkability Composite Index (6) and the Rate Stabilizing Tool (RST) (11). The Peel Walkability Composite Index uses a diverse range of measures to construct a repeatable measure of neighborhood walkability. The RST responds to the demand for high-quality, local-level estimates of chronic disease, and enables users with limited statistical expertise to generate reliable local-level, age-standardized, and spatially smoothed measures of chronic disease.

The rapid expansion of geo-referenced data sets is a critical driver of the increasing numbers and sophistication of geo-spatial studies. This collection includes the use of geo-referenced data from electronic health records (2), emergency medical services (EMS) (15), and market research (8). These large geo-referenced data sets have the potential to provide important insights into the geographic patterns and drivers of chronic diseases. One study demonstrates the novel application of a widely used, publicly available geo-referenced data source — Google Street View — for public health promotion (5). 

A key element in conducting geospatial studies is striking the balance between the presentation of local-level data at the smallest appropriate geographic unit and the limitations of generating robust estimates in the presence of small population sizes and numbers of health outcomes. The studies in this collection have all successfully navigated this tension and present data across multiple geographic levels (census tract [6,9,15,16], county [10,14,17,18], and locally meaningful definitions of neighborhood [8,12]) with results that are statistically reliable and meaningful to stakeholders. One study developed a spatial statistical approach to overcoming some of the limitations of data that are highly censored for confidentiality reasons, thereby enabling state and local health departments to generate small area estimates using publicly available data (10).

Recognizing the potent communication capacity of maps, several articles in this collection explore novel geospatial visualizations that may supplement more commonly used maps and report data in an approachable and actionable format. For example, ring maps (19) allow the simultaneous visualization of multiple measures related to chronic diseases. Other studies include figures that demonstrate changes in hotspots over time, allowing a single figure to overcome the limitations of typical cross-sectional maps (12). Visualizing spatial data has also allowed first responders to identify patients at risk during a natural disaster (20) and allowed public institutions to collaborate with health systems, community organizations, and the public to use geospatial data to improve public health and address health equity in birth outcomes (20). Many of the studies published in this collection have also used the Chronic Disease GIS Snapshot article type, unique to *Preventing Chronic Disease* (21). GIS Snapshots are brief reports that focus on using maps to communicate the extent of geographic disparities in chronic disease–related outcomes and risk factors with an eye to providing information for guiding chronic disease prevention programs and policies.

## Using Maps and Geospatial Results to Guide Program and Policy Decisions

Another key theme in this special collection is the use of geospatial data to inform programs and policies for chronic disease prevention and treatment. For example, the authors of a study about walkability state that, “Understanding the capacity of the built environment to facilitate walking for utilitarian purposes allows public health departments to advocate for strategic land use and infrastructure developments that promote an increase in population physical activity levels” (6). Several studies in this collection document geographic disparities in access to care (eg, for chronic disease management [22], blood pressure medication adherence [17], diabetes prevention programs [18], and asthma prevention programs [1,12]), providing compelling guidance about where facilities and services are needed. A unique study demonstrates the use of real-time GIS to develop and update emergency response for chronically ill veterans during Hurricane Irma (23). From an applied perspective, staff members from 4 health departments (Maine Center for Disease Control and Prevention, New Jersey Department of Health, New York State Department of Health, and Cuyahoga County, Ohio, Board of Health) describe the ways in which GIS has become a critical tool (24). Their article provides specific examples of how health departments use maps and spatial analyses to 1) communicate the burden of disease; 2) inform decisions about resource allocation, policy, and priority communities for intervention efforts; 3) develop culturally competent programs; and 4) assist with program planning, monitoring, and evaluation.

By embracing the benefits of GIS, increasing the volume of spatially referenced public health data, and applying a broad range of spatial statistical tools, public health practitioners and investigators are continually pushing the envelope for using geospatial data to inform surveillance, epidemiologic research, program evaluation, resource allocation, and communication for chronic disease prevention and treatment. We invite readers to engage deeply with the geospatial approaches presented in this special collection, to contemplate further advances in understanding how place and space shape the distribution of chronic diseases, and to apply a geospatial perspective to promote health equity and inform public health action for chronic disease prevention and treatment.
